# The Skin–Brain–Exposome Axis in Stress-Sensitive Dermatoses: A Narrative Review

**DOI:** 10.3390/jcm15083036

**Published:** 2026-04-16

**Authors:** Anna Kubrak, Siddarth Agrawal, Mateusz Dróżdż, Jacek C. Szepietowski, Jarosław Dybko

**Affiliations:** 1 Labplus R&D, Wyspa Słodowa 7, 50-266 Wroclaw, Poland; a.kubrak@labplus.pl (A.K.); m.drozdz@labplus.pl (M.D.); 2 Division of Dermatology, Venereology and Clinical Immunology, Faculty of Medicine, Wroclaw University of Science and Technology, 50-386 Wroclaw, Poland; jacek.szepietowski@pwr.edu.pl; 3 Department of Dermato-Venereology, 4th Military Hospital, 50-981 Wroclaw, Poland; 4 Department of Oncology and Hematology, Wroclaw University of Science and Technology, 50-370 Wrocław, Poland; jaroslaw.dybko@pwr.edu.pl

**Keywords:** skin–brain axis, exposome, psychoneuroimmunology, psychodermatology, atopic dermatitis, psoriasis, chronic pruritus, cognitive behavioral therapy

## Abstract

**Background**: Dermatological conditions represent a leading cause of global nonfatal disease burden, accounting for approximately 42.9 million disability-adjusted life years annually. Their complex pathogenesis is increasingly understood through the skin–brain–exposome axis, a bidirectional neuroimmunological and environmental communication network. The study aims to synthesize the neurobiological mechanisms of the skin–brain–exposome axis with macroscopic sociodemographic modifiers, clinical manifestations, and evidence-based psychodermatological interventions. **Methods**: A narrative review was conducted, following a structured search of PubMed, Scopus, and Web of Science (from inception to February 2026), yielding 54 sources. Mechanistic and interventional data (including randomized controlled trials and meta-analyses) were integrated with large-scale population-based epidemiological findings, anchored by a recent cross-sectional Polish cohort of 27,000 adults. **Results**: Psychological distress is associated with hyperactivation of the hypothalamic–pituitary–adrenal (HPA) axis and peripheral neurogenic inflammation (e.g., Substance P, corticotropin-releasing hormone), exacerbating stress-sensitive conditions such as atopic dermatitis, psoriasis, acne, and chronic pruritus. External exposome factors (urbanization, pollution) and sociodemographic variables (education, gender) may modify biological risk and diagnostic capture rates, frequently generating an epidemiological diagnostic paradox. Randomized trials support that psychotherapeutic interventions, particularly Cognitive Behavioral Therapy (CBT) and Mindfulness-Based Stress Reduction (MBSR), effectively disrupt the physical itch–scratch–stress cycle and improve disease-specific quality of life, serving as evidence-based adjunctive strategies in comprehensive care. **Conclusions**: Effective dermatological management requires targeting both the cutaneous barrier and the psychological exposome. Integrating routine psychosocial screening and stratified behavioral interventions into standard clinical care is essential for addressing the neuroimmune chronicity of inflammatory skin diseases.

## 1. Introduction

Epidemiological data demonstrates that skin diseases represent a substantial global public health burden, severely impacting health-related quality of life. According to the Global Burden of Disease (GBD) Study 2019, dermatological conditions consistently rank among the leading causes of nonfatal disease burden worldwide, accounting for approximately 42.9 million disability-adjusted life years (DALYs) annually [1]. Notably, this burden continues a trend from previous GBD estimates where skin diseases are already ranked as the fourth leading cause of nonfatal burden globally—exceeding conditions such as diabetes mellitus and migraines. Transitioning from the global perspective to the European context, recent population-based surveys estimate that over 185 million individuals across Europe are affected by at least one dermatological condition, with more than 94 million Europeans experiencing uncomfortable skin sensations such as pruritus and burning [2].

However, broad regional estimates often mask the extent of localized disease burden. A recent, large-scale cross-sectional analysis of 27,000 adults in Poland demonstrated an 89.5% lifetime prevalence of at least one physician-diagnosed skin disease among the surveyed adult population [3]. This high prevalence highlights dermatological health as a primary public health priority.

To understand the complex etiology and exacerbation of these widespread conditions, contemporary dermatology increasingly embraces two highly interrelated conceptual frameworks: the “Skin–Brain Axis” and the “Skin Exposome.” The skin–brain axis is defined as a complex, bidirectional neuroimmunological communication network linking the central nervous system (CNS) and the skin [4,5]. Through this neuroendocrine axis, psychological distress is frequently associated with the exacerbation of cutaneous inflammation, while cutaneous distress can reciprocally signal the brain to alter neurochemistry and mood [6,7,8]. Concurrently, the “Skin Exposome” conceptualizes the totality of environmental, lifestyle, and behavioral exposures an individual encounters throughout their lifespan, encompassing external factors like allergens and microorganisms, as well as internal psychosocial stressors [9,10].

These overarching frameworks integrate seamlessly; environmental insults from the exposome may compromise skin barrier integrity and trigger peripheral neuroimmune responses. These responses subsequently communicate with the brain, modulating systemic stress responses that can further modify an individual’s behavioral interactions with their environment [11,12]. Despite the profound clinical implications of these interconnected systems, a structural gap remains in the scientific literature. Current dermatological and neuroimmunological research extensively maps individual molecular mechanisms or catalogs sociodemographic risk factors through descriptive epidemiology. However, the literature rarely integrates both the intricate neurobiological mechanisms and the macroscopic environmental and sociodemographic determinants into a unified, population-grounded framework.

This narrative synthesis aims to bridge this knowledge gap. By constructing a holistic, biopsychosocial model of skin health, this review will outline the epidemiological burden of skin diseases, explore the molecular and cellular mechanisms of the bidirectional skin–brain axis, and analyze the cumulative impacts of the skin exposome. Furthermore, it will explore how effective stress coping mechanisms are significantly associated with a reduced prevalence of six common dermatological manifestations (pruritus, burning, redness, rash, desquamation, and sunburn), as demonstrated in the recent Polish cohort (*p* < 0.001) [3]. It is explicitly noted that this document serves as a comprehensive narrative synthesis and conceptual integration, rather than a systematic review.

## 2. Materials and Methods

Although this manuscript is structured as a narrative synthesis and conceptual integration, a structured literature search strategy was employed to ensure rigor and mitigate selection bias. An electronic literature search was conducted across three primary databases: PubMed/MEDLINE, Scopus, and Web of Science. The search encompassed literature published from inception up to February 2026, with a prioritized focus on high-impact publications from 2018 to 2026 to capture the most contemporary neuroimmunological and exposome data.

The search strategy utilized a combination of Medical Subject Headings (MeSH) and free-text keywords, structured around three core domains: (1) Dermatological conditions (“Atopic Dermatitis” OR “Eczema” OR “Psoriasis” OR “Acne Vulgaris” OR “Rosacea” OR “Pruritus” OR “Alopecia”); (2) Psychoneuroimmunology (“Skin-Brain Axis” OR “Psychological Stress” OR “Neurogenic Inflammation” OR “HPA Axis” OR “Neuropeptides”); and (3) Environmental/Interventional factors (“Exposome” OR “Urbanization” OR “Mindfulness” OR “Cognitive Behavioral Therapy” OR “Lifestyle”).

To construct the clinical and interventional sections (Results: Section 4), selection criteria strictly prioritized randomized controlled trials (RCTs), systematic reviews, and meta-analyses. For the epidemiological and exposome sections (Results: Section 3.1 and Section 3.2), large-scale population-based cross-sectional studies and prospective cohorts were prioritized. Case reports and non-peer-reviewed patient organization literature were excluded. Following the removal of duplicates, articles were screened by title and abstract for relevance to the biopsychosocial model of dermatology, and the final reference library was synthesized to form the foundation of this review.

## 3. Results

### 3.1. Neuroimmunological Mechanisms of the Skin–Brain Axis

Recent large-scale epidemiological investigations have broadened the boundaries of clinical dermatology, transitioning from a discipline focused purely on localized cutaneous pathology to one that accounts for systemic, psychoneuroimmunological integration. The skin and the central nervous system (CNS) share a lifelong connection derived from their common embryonic origin in the ectoderm, establishing the anatomical foundation for the bidirectional skin–brain axis [4,5]. Advancing dermatological science requires moving beyond observational epidemiology to elucidate the plausible molecular pathways that connect cognitive stress appraisal to cellular cutaneous phenotypes [11].

#### 3.1.1. HPA Axis Dysregulation and Skin Barrier Dysfunction

The hypothalamic–pituitary–adrenal (HPA) axis serves as the primary systemic physiological responder to psychological distress [5]. Upon exposure to chronic stress, the hypothalamus secretes corticotropin-releasing hormone (CRH), which ultimately triggers the secretion of glucocorticoids, prominently cortisol, from the adrenal glands. Crucially, human skin possesses a fully functional, localized peripheral HPA-like equivalent [4,11]. Epidermal keratinocytes, melanocytes, and sebocytes are capable of synthesizing CRH and locally producing cortisol in response to environmental or psychological stressors [5,13].

The mechanistic literature indicates that prolonged elevation in both systemic and localized cortisol exerts adverse effects on epidermal barrier function [11]. Glucocorticoids inhibit the synthesis of crucial epidermal lipids, including ceramides, while simultaneously suppressing keratinocyte proliferation and differentiation [14]. This stress-induced disruption of the stratum corneum leads to increased transepidermal water loss (TEWL) and clinical desquamation.

These established mechanistic pathways offer a plausible biological interpretation for population-level observations. A large-scale Polish cohort demonstrated an 89.5% prevalence of at least one physician-diagnosed skin disease among adults [15]. Furthermore, the cohort data revealed that the deployment of effective psychological stress coping mechanisms was significantly associated with a reduced prevalence of desquamation (*p* < 0.001) [3]. While cross-sectional survey methodologies inherently preclude causal inferences regarding epidermal cortisol levels or lipid synthesis, existing psychoneuroimmunological models suggest that adaptive stress coping may dampen systemic HPA axis hyperactivation, potentially mitigating the downstream barrier dysfunction.

#### 3.1.2. Neurogenic Inflammation

In addition to neuroendocrine HPA pathways, the skin–brain axis is mediated by direct innervation of the epidermis and dermis by unmyelinated afferent C-fibers and autonomic nerves [8]. Psychological stress triggers different neural signaling that impacts cutaneous immunology through a process known as neurogenic inflammation. Upon physiological or psychological stimulation, these peripheral nerve endings release neuropeptides, most notably Substance P (SP), nerve growth factor (NGF), and calcitonin gene-related peptide (CGRP) [6,8].

Substance P exhibits a high binding affinity for neurokinin-1 (NK-1) receptors located on the surface of cutaneous mast cells, which are notably localized in close proximity to peripheral nerves in the skin [12]. The binding of SP to NK-1 triggers non-IgE-mediated mast cell degranulation, releasing a cascade of pro-inflammatory mediators including histamine, tryptase, and tumor necrosis factor-alpha (TNF-α) [8,16]. Concurrently, CGRP acts as a vasodilator, increasing localized dermal blood flow and microvascular permeability [5]. Clinically, the release of histamine and tryptase drives pruritus, while CGRP-induced vasodilation manifests as erythema and burning sensations.

The theoretical downregulation of these neurogenic pathways via psychological intervention is supported by robust clinical trial data. In a randomized clinical trial (N = 107) of adults with atopic dermatitis, integrated online mindfulness and self-compassion training significantly improved disease-specific quality of life compared to standard care, showing a substantial between-group difference on the Dermatology Life Quality Index (DLQI) at 13 weeks (−6.34; 95% CI, −8.27 to −4.41; *p* < 0.001) with a large standardized effect size (Cohen’s d = −1.06) [17]. Similarly, an RCT (N = 102) evaluating internet-delivered Cognitive Behavioral Therapy (CBT) demonstrated a moderate-to-large significant reduction in eczema symptoms on the Patient-Oriented Eczema Measure (POEM) (d = 0.75; 95% CI, 0.32–1.16; *p* < 0.001) [18]. These interventional findings provide a high-level evidentiary counterpart to the observational data from the Polish cohort, wherein effective stress coping was associated with decreased odds of experiencing pruritus, redness, and burning sensations (*p* < 0.001) [3].

#### 3.1.3. Immune Dysregulation and Cytokine Pathways

The downstream consequence of prolonged HPA activation and neurogenic inflammation is complex immunological dysregulation [19]. Chronic psychological stress is demonstrated to skew the adaptive immune response, frequently altering the T-helper 1 (Th1) and T-helper 2 (Th2) balance, while also upregulating Th17 pathways [5,20]. In atopic diatheses, stress exacerbates the Th2 inflammatory axis. Stress-induced neuroimmunological signals stimulate the overproduction of specific interleukins, most critically IL-31, widely designated as the “itch cytokine” [8]. IL-31 directly bridges the immune and nervous systems by binding to receptors on sensory neurons, creating a central and peripheral sensitization loop that further damages the compromised epidermal barrier [16].

Conversely, in conditions like psoriasis, psychological stress influences the Th17/IL-23 axis [19]. Elevated levels of IL-17, IL-22, and TNF-α, driven by psychoneuroimmunological stress signaling, stimulate keratinocyte hyperproliferation, manifesting clinically as erythematous, scaly plaques [20]. The integration of these specific cytokine pathways offers a mechanistic context for population-level observations. In the Polish cohort, positive lifestyle indicators and effective stress mitigation were correlated with a decreased incidence of rashes and persistent pruritus [3]. This association may be consistent with the hypothesis that dampening the initial neuroendocrine distress signal helps maintain the delicate equilibrium of Th1/Th2 and Th17 responses, averting the overproduction of the cytokines driving these clinical symptoms.

#### 3.1.4. Evidence Gaps Identified

Lack of Longitudinal and Causal Human Data:

While cross-sectional epidemiological studies [3] robustly establish statistical associations between psychosocial factors and dermatological symptoms, they cannot establish causation. There is a critical need for longitudinal cohort studies that concurrently track psychological stress indices and objective cutaneous biomarkers (e.g., epidermal cortisol, TEWL, serum cytokines) over time in human populations.

Translation from Animal to Human Models:

Many foundational mechanisms of neurogenic inflammation—such as specific SP and CGRP neural circuits—have been mapped primarily in murine models [11]. The direct translation of these precise neuro-immune interactions to the complex human psychosocial environment remains incomplete, necessitating advanced in vivo human neuroimaging or continuous biomarker monitoring during validated psychological stress protocols.

Sociodemographic Nuance in Pathomechanisms:

Current neuroimmunological research rarely accounts for macroscopic variables. It remains unclear how sociodemographic factors, such as educational attainment or urbanization, biologically interact with or modify the intrinsic sensitivity of the HPA axis or peripheral neurogenic pathways.

### 3.2. The Impact of Psychological Stress on Core Dermatoses

Building upon the fundamental neuroimmunological mechanisms of the skin–brain–exposome axis outlined above, the following subsections systematically evaluate how these pathways manifest clinically across core stress-sensitive dermatoses.

Epidemiological anchoring for the profound impact of this axis is robustly supported by a recent cross-sectional population study conducted in Poland (N = 27,000; comprising 22,043 women and 4887 men) [15]. This large-scale analysis revealed a staggering public health burden, demonstrating that 89.5% of adults report the presence of at least one concurrent skin disease. The reported prevalences of specific, immune-mediated conditions within this cohort were exceptionally high: atopic dermatitis/eczema affected 25.5%, psoriasis 5.0%, acne vulgaris 32.7%, and hair loss 34.8% of the population [15].

Crucially, this study demonstrated that self-reported effective stress coping capabilities were significantly associated with a marked reduction in the prevalence of six core dermatological manifestations: pruritus, burning sensations, redness, rash, desquamation, and sunburn (*p* < 0.001 for all parameters) [3]. The data also highlighted notable sociodemographic variances; women reported significantly more frequent erythema and burning sensations, whereas younger demographics (18–24 years) exhibited increased instances of rash and desquamation compared to older cohorts.

Strict methodological guardrails require acknowledging that cross-sectional epidemiological associations, such as those observed in the Polish cohort, provide vital population-level context but inherently preclude the direct inference of longitudinal causality. Cross-sectional data capture point-in-time correlations; thus, while effective stress coping is robustly associated with reduced symptom prevalence, causality regarding the initial onset of these disease states requires prospective validation. Furthermore, it is imperative to maintain a strict delineation between symptom-level evidence (e.g., the acute exacerbation of pruritus or transient erythema) and disease-level evidence (e.g., the fundamental chronicity or initial pathophysiological onset of the dermatosis).

The following subsections systematically evaluate the primary stress-related pathomechanisms, the evidence grading for specific clinical manifestations, and the severe bidirectional psychological burdens inherent to core dermatological conditions, utilizing the contemporary literature.

#### 3.2.1. Atopic Dermatitis/Eczema

Atopic dermatitis (AD), commonly referred to as eczema, is a chronic, highly heterogeneous inflammatory skin disorder characterized by a defective epidermal barrier, severe pruritus, and localized immune dysregulation. Within the Polish study cohort, AD represented a massive burden, affecting 25.5% of the adult population [15]. Patients suffering from AD frequently present with acute symptom flares that population-level data suggest are strongly mitigated by the deployment of effective stress coping mechanisms [3]. The key pathophysiological and clinical domains of AD, which are further detailed in the following subsections, are summarized in Table 1.

- Primary Stress-Related Pathomechanism

The pathogenesis of stress-induced exacerbation in AD is closely linked to the cutaneous neuroendocrine system and the systemic activation of the HPA axis. Studies suggest that acute and chronic psychological stress can precipitate the release of corticotropin-releasing hormone (CRH) and Substance P (SP) from both central pathways and peripheral cutaneous nerve terminals [5,11]. In the localized environment of the skin, these neuropeptides may directly stimulate mast cell degranulation and upregulate the expression of T-helper 2 (Th2) and Th17 cytokines, most notably interleukin (IL)-4, IL-13, and IL-31 [5]. This critical cytokine shift drives neurogenic inflammation and further compromises the structural integrity of the stratum corneum. Clinically, AD manifests as poorly defined erythematous patches, often accompanied by exudative lesions (weeping) in acute stages, or lichenification (thickening of the skin) and severe excoriations resulting from chronic scratching [11,14].

Furthermore, the internal exposome, specifically the gut–skin axis, plays an adjunctive role in AD and psoriasis pathology [21]. Psychological stress may alter intestinal mucosal permeability, leading to microbial dysbiosis. The depletion of short-chain fatty acid (SCFA)-producing bacteria in the gut can compromise systemic immune tolerance, facilitating the dissemination of pro-inflammatory mediators [10]. Environmental exposome factors interact adversely with stress; continuous exposure to environmental pollutants (such as PM2.5 and NO2) combined with early-life psychological stress has been associated with an increased risk of childhood AD, supporting a “(pre-)fetal origin of eczema” hypothesis [10].

- Clinical Manifestations Impacted by Distress

Psychological distress is frequently associated with the exacerbation of subjective symptom severity in AD, particularly the intensity of pruritus, the architectural destruction of sleep, and the expansion of visible eczematous lesions. Experimental models indicate that chronic stress may weaken the skin barrier by elevating basal cortisol, which impairs lipid synthesis and leads to heightened transepidermal water loss [11].

Regarding evidence grading, there is robust evidence that stress acts as an amplifier for AD symptom flare-ups. Meta-analytical data indicates that while AD patients have high rates of mental health symptoms, the baseline clinical severity of AD (as measured by objective scoring systems like SCORAD) is not always linearly correlated with the lifetime prevalence of mental health disorders [22]. This supports the interpretation that psychological stress acts more potently as an acute symptom amplifier rather than the sole arbiter of lifelong disease severity.

- Bidirectional Relationship: Psychological Burden and Stigma

The relationship between atopic dermatitis and psychological distress is relentlessly cyclical and profoundly impacts patient quality of life [23,24]. The visible nature of eczematous rashes and the socially disruptive urge to scratch frequently cause profound social stigmatization and withdrawal. Systematic reviews demonstrate that adults and adolescents with AD exhibit a 20% to 30% higher baseline prevalence of clinical depression, anxiety disorders, and suicide-related behaviors compared to the healthy general population [25].

This bidirectional pathology is defined by the “itch–scratch–stress” cycle. The physiological urge to scratch disrupts sleep architectures, and the resulting sleep deprivation may elevate systemic cortisol levels, further impairing skin barrier maintenance [11]. The necessity of targeted psychodermatological interventions to break this cycle is supported by robust quantitative clinical data. A recent randomized clinical noninferiority trial (N = 168) evaluating brief online cognitive–behavioral therapy (CBT) for AD demonstrated a clinically significant reduction in symptom severity, with patients improving by a mean of 4.20 to 4.60 points on the Patient-Oriented Eczema Measure (POEM) post-intervention [26]. Similarly, another RCT (N = 107) found that integrated mindfulness and self-compassion training significantly reduced disease-specific impairment, yielding a large standardized effect size (Cohen’s d = −1.06) for quality of life improvements at 13 weeks [17].

**Table 1:** Pathophysiological and clinical domain in atopic dermatitis.

Domain	Summary Data
Primary Pathomechanism	HPA axis dysfunction; neuropeptide (SP, CRH) release associated with mast cell degranulation; Th2/Th17 cytokine shift (IL-4, IL-13, IL-31); gut microbiome dysbiosis reducing systemic SCFA production.
Symptom-Level Impact	Acute exacerbation of pruritus intensity, localized desquamation, transient erythema, and severe disruption of sleep architecture.
Disease-Level Impact	Associated with the exacerbation of overall lesion surface area; hypotheses link maternal stress and environmental pollutants (PM2.5) to childhood disease onset.
Evidence Grade	Strong evidence for symptom amplification and sleep disturbances during periods of acute stress.
Bidirectional Burden & Stigma	Severe stigmatization from visible scratching; 20–30% increased prevalence of clinical anxiety/depression; persistent, self-sustaining itch–scratch–stress cycle.

#### 3.2.2. Psoriasis

Demonstrating a significant population footprint, the prevalence of psoriasis in the adult Polish cohort was documented at 5.0% [15]. Unlike the Th2 dominance typically seen in atopic dermatitis, the stress-related pathomechanism in psoriasis appears heavily mediated by the Th17/IL-23 immune axis [19].

- Primary Stress-Related Pathomechanism

Psychoneuroimmunological stress signaling is hypothesized to trigger a cascade wherein peripheral nerve endings release neuropeptides that stimulate dendritic cells and macrophages [2,5]. This neuro-immune interaction is associated with the upregulation of Interleukin-17 (IL-17), IL-22, and tumor necrosis factor-alpha (TNF-α) [19,20]. Elevated IL-17 is a primary biological driver of excessive keratinocyte hyperproliferation and altered differentiation, which clinically manifests as the thick, erythematous, and scaly plaques characteristic of psoriasis [27,28]. These biological processes result in the characteristic clinical phenotype of well-demarcated, erythematous plaques covered with silvery-white micaceous scales, typically localized on extensor surfaces [4,19].

- Bidirectional Relationship and Psychological Burden

The bidirectionality of the skin–brain axis is clinically significant in psoriasis. Due to the chronic, highly visible, and frequently stigmatized nature of psoriatic plaques, patients experience disproportionately high rates of secondary psychiatric comorbidities, including major depressive disorder, social withdrawal, and severe anxiety [4,19]. This psychosocial distress acts as a systemic environmental stressor, continuously triggering the neuroendocrine dysregulation that can fuel the Th17 inflammatory cascade, thereby creating a perpetuating psychodermatological cycle.

- Quantitative Intervention Evidence

The hypothesis that mitigating psychological stress can biologically dampen this inflammatory cascade is supported by robust quantitative data. A recent meta-analysis by Wei et al. (2024) analyzing 1048 subjects demonstrated that psychological interventions significantly improved anxiety symptoms in patients with psoriasis (Standardized Mean Difference [SMD] = −0.41; 95% CI, −0.77 to −0.05; *p* = 0.001) [29]. Similarly, a systematic review and meta-analysis by Zill et al. (2019) evaluating psychosocial interventions (primarily cognitive behavioral and mindfulness-based techniques) across 19 studies found significant, small-to-medium positive effects on both quality of life (SMD = 0.28; 95% CI, 0.04 to 0.51; n = 664) and anxiety (SMD = −0.36; 95% CI, −0.57 to −0.15; n = 363) [30]. These findings strongly suggest that targeted cognitive restructuring provides quantifiable relief from the psychosocial burdens that exacerbate psoriatic flares.

#### 3.2.3. Acne Vulgaris and Rosacea

Acne vulgaris represents one of the most widespread dermatological conditions, with a high lifetime prevalence of 32.7% identified within the adult Polish cohort [15]. Both acne and rosacea possess strong connections to the internal and external exposome, particularly regarding stress and diet.

- Primary Stress-Related Pathomechanism

The physiological stress response has been linked to alterations in the pilosebaceous unit, which is heavily innervated and expresses receptors for various neuroendocrine mediators [5]. During periods of psychological distress, the localized cutaneous HPA-like axis is activated, prompting the local production of corticotropin-releasing hormone (CRH) in sebocytes. This local CRH acts as a potent lipogenic stimulus, driving sebum overproduction. When combined with stress-induced follicular hyperkeratinization and the localized cutaneous exposome—such as dietary factors like high glycemic index nutrition, which elevates IGF-1—this process facilitates the formation of comedones and inflammatory papulopustular lesions [31]. Acne vulgaris is characterized by the presence of open and closed comedones, inflammatory papules, and pustules, predominantly in seborrheic areas. In contrast, rosacea primarily presents as persistent centrofacial erythema, telangiectasias (visible blood vessels), and episodes of transient flushing [31,32].

In parallel, conditions like rosacea are frequently exacerbated by stress-induced neurogenic inflammation. The release of potent vasodilators, such as calcitonin gene-related peptide (CGRP), from afferent nerve endings provokes microvascular dilation, potentially contributing to acute facial erythema and flushing [5].

- Bidirectional Relationship and Interventional Evidence

Given the facial predominance of both acne and rosacea, the bidirectional psychological impact is severe. For rosacea specifically, recent Mendelian randomization analyses suggest a potentially bidirectional relationship with psychiatric disorders, though clinical interpretation remains evolving [32]. The appearance of visible facial lesions has been shown to correlate with altered self-perception, generating internalized stigma, social anxiety, and psychological morbidity [33,34] that, in turn, translates into further peripheral CRH release and localized sebaceous lipogenesis.

The integration of psychological and exposome-based interventions shows promising clinical efficacy in breaking this cycle. A randomized, two-armed experimental trial (N = 30) investigating a novel cognitive stress management technique (Pythagorean Self-Awareness Intervention) in adult female patients with acne vulgaris found that 93.3% of patients in the intervention group showed an improvement in clinical acne stage over 8 weeks, compared to only 26.7% in the control group (Relative Risk: 3.5; 95% CI, 1.5 to 8.2; *p* = 0.001) [35]. Furthermore, addressing the external microbial exposome also yields quantifiable results; an RCT (N = 20) utilizing a topical probiotic (*Enterococcus faecalis* lotion) to target microbiome dysbiosis demonstrated significant improvements in investigator-assessed acne severity scores after just 2 weeks (*p* = 0.009) and 6 weeks (*p* < 0.0005) [36]. These findings, while limited by small sample sizes, provide preliminary proof-of-concept for exposome-targeted therapies and warrant replication in larger, powered trials. Together, these data underscore the necessity of managing both the psychological and microbial exposome to effectively treat acne vulgaris.

#### 3.2.4. Hair Loss (Alopecia)

Hair loss represents a pervasive dermatological complaint, affecting 34.8% of the adult population in the Polish cohort [15]. It is important to note that this cross-sectional survey captured generalized hair loss rather than distinguishing between specific clinical subtypes (e.g., alopecia areata vs. telogen effluvium). Therefore, while the survey establishes a high population-level burden, specific mechanistic interpretations must rely on external psychoneuroimmunological models.

- Primary Stress-Related Pathomechanisms

The mammalian hair follicle is a complex mini-organ whose cyclical growth is highly susceptible to neuroendocrine fluctuations [5]. Acute or chronic psychological distress is hypothesized to disrupt the normal anagen (growth) phase of the hair cycle. Systemic elevations in cortisol, alongside the local release of Substance P (SP) and corticotropin-releasing hormone (CRH), may promote premature apoptosis of hair follicle keratinocytes, potentially accelerating the follicle’s shift into the telogen (resting and shedding) phase. Depending on the subtype, this may manifest as diffuse hair thinning across the scalp or the appearance of circumscribed, smooth bald patches in autoimmune variants like alopecia areata [6].

In autoimmune-mediated variants, such as alopecia areata (AA), stress-induced neurogenic inflammation is theorized to trigger a collapse of the hair follicle’s physiological “immune privilege” [6]. This collapse may expose normally hidden follicular autoantigens to the systemic immune system, theoretically facilitating targeted, immune-mediated destruction of the follicle.

- Bidirectional Relationship and Psychological Interventions

The psychosocial sequelae of hair loss are clinically significant. Because hair is deeply intertwined with human identity, gender expression, and social signaling, sudden or progressive alopecia represents a profound threat to self-esteem and body image. The emotional distress and identity disruption caused by shedding act as systemic stressors, which may fuel further neurogenic inflammation and immune privilege collapse. Recognizing this severe psychodermatological burden, clinical frameworks are increasingly integrating colocated behavioral health care into dermatology clinics to address the secondary psychiatric comorbidities of conditions like AA, aiming to structurally disrupt this stress–shedding cycle [37].

#### 3.2.5. Chronic Pruritus

While not a standalone disease entity, chronic pruritus is a ubiquitous, transdiagnostic symptom whose severity is inextricably linked to psychological stress and complex pathogenic pathways [8,38,39]. In the Polish cohort, effective stress coping mechanisms were strongly associated with a decreased prevalence of persistent pruritus (*p* < 0.001) [3].

- Primary Stress-Related Pathomechanism

Across multiple dermatoses, psychological distress has been shown to amplify the sensation of itch through the overproduction of specific pro-pruritic mediators, most critically Interleukin-31 (IL-31) and Substance P [8]. When the HPA axis and autonomic nervous system are hyperactivated, these molecules bind to specialized receptors (such as neurokinin-1) located on unmyelinated C-nerve fibers in the skin. This initiates an intense afferent neurogenic signal to the somatosensory cortex, cognitively interpreted as severe itch, while simultaneously activating reward circuits in the brain upon scratching [16].

The bidirectionality of chronic pruritus manifests as the “itch–scratch–cycle.” The neurological sensation of pruritus drives the mechanical act of scratching, which physically excoriates the stratum corneum. This barrier trauma provokes secondary inflammation and the release of further pruritogens. Clinically, this manifests as visible linear excoriations (scratch marks), hemorrhagic crusts, and, in chronic cases, lichen simplex chronicus (thickened, leathery skin) or prurigo nodules resulting from repetitive mechanical trauma to the epidermal barrier [38,39]. Simultaneously, the physical sensation of chronic itch severely disrupts sleep architecture and daily cognitive function, inducing psychological exhaustion that biologically primes the nervous system for heightened pruriceptive signaling [8,16].

- Quantitative Intervention Evidence

Targeting the psychological component of the itch–scratch cycle has yielded quantifiable clinical success. A recent comprehensive meta-analysis of 20 randomized controlled trials evaluating psychological interventions across various chronic itch conditions found significant post-treatment reductions in self-rated itch intensity (Hedges’ g = −0.37; 95% CI, −0.60 to −0.15), externally rated excoriations (g = −0.29; 95% CI, −0.49 to −0.09), and self-rated scratching intensity (g = −0.99; 95% CI, −1.35 to −0.63) [40].

Furthermore, even alternative sensory and relaxation interventions aimed at stress reduction demonstrate measurable efficacy. An open randomized prospective trial (N = 50) evaluating the impact of a music relaxation intervention on patients with chronic pruritic skin diseases demonstrated a significantly greater reduction in acute pruritus intensity in the intervention group compared to standard emollient care (mean reduction of 2.3 vs. 1.2 on a Numerical Rating Scale; *p* < 0.05) [41]. These findings are directionally consistent with the epidemiological observation that structured stress mitigation serves as a highly effective, adjunctive therapeutic tool for managing transdiagnostic pruritus.

## 4. Sociodemographic and Environmental Modifiers of Skin Health

The external skin exposome represents the cumulative, highly dynamic totality of environmental, psychosocial, and sociodemographic exposures an individual encounters throughout their lifespan. These factors continuously interact with the neuroendocrine and immune systems to modulate dermatological health [10]. Analyzing this intricate web requires robust epidemiological data to identify associations while carefully decoupling confounding variables.

Recent evidence derived from the nationwide cross-sectional study in Poland (N = 27,000) underscores the ubiquitous nature of this burden, with 89.5% of the adult population reporting at least one physician-diagnosed skin disease [15]. However, the individual response to neuroimmune stressors is not uniform; it is heavily modified by the broader macro-environment and sociodemographic status of the individual. Integrating these cross-sectional findings with the global epidemiological literature reveals distinct patterns in how urbanization, educational attainment, and sex/gender disparities are associated with dermatological risk profiles and diagnostic capture rates (summarized in Table 2).

### 4.1. Urbanization and Pollution: Environmental Determinants

Global urbanization trends have fundamentally altered the ambient environment, subjecting the cutaneous barrier to atmospheric pollutants, including particulate matter (PM2.5 and PM10), volatile organic compounds (VOCs), and ground-level ozone. The epidemiological consequences of this exposure are reflected in distinct urban–rural disease gradients.

The Polish population study determined that residents of large cities (exceeding 500,000 inhabitants) experienced significantly higher odds of developing atopic dermatitis (OR = 1.20) compared to those in smaller towns and rural areas [15]. The pathophysiology underpinning this urban gradient is heavily linked to the activation of the aryl hydrocarbon receptor (AhR) pathway. Urban pollutants, specifically polycyclic aromatic hydrocarbons (PAHs), act as exogenous ligands for the AhR [10]. Chronic activation of this pathway by urban smog triggers localized oxidative stress, depletes stratum corneum antioxidants, and upregulates pro-inflammatory mediators, thereby compromising barrier function and exacerbating Th2-mediated inflammatory cascades.

Furthermore, the dataset revealed a twofold increase in the risk of non-melanoma skin cancer (NMSC) among residents of large cities (OR = 2.00) [15]. While ultraviolet (UV) radiation unequivocally remains the primary driver of NMSC, particularly among outdoor workers [42,43], urban atmospheric pollutants may act as co-carcinogens. In-depth molecular models suggest that cutaneous Langerhans cells may metabolically convert environmental PAHs into reactive intermediates that synergize with direct UV-induced DNA damage. However, this pronounced epidemiological spike (OR = 2.00) must also be viewed through the critical methodological lens of healthcare access. Large urban centers house the highest density of specialized dermatological clinics and advanced diagnostic technologies. Consequently, the urban NMSC spike likely represents a composite: a biological vulnerability magnified by significantly elevated diagnostic capture rates among urban populations with ready access to specialist screening.

### 4.2. Education, Health Literacy, and the Diagnostic Paradox

In generalized epidemiological models, higher educational attainment often inversely correlates with disease morbidity due to improved health literacy and lower rates of hazardous lifestyle behaviors. However, when analyzing dermatological epidemiology, educational attainment frequently yields a complex phenomenon termed the “Diagnostic Paradox”.

In the Polish cohort, higher educational attainment was associated with a statistically significant increase in the prevalence of both acne vulgaris (OR = 1.50) and NMSC (OR = 1.40) [15]. It is biologically unlikely that advanced education directly causes comedogenesis or epidermal mutagenesis. Rather, this association likely reflects ascertainment bias. Highly educated individuals generally possess greater health literacy and financial resources, resulting in proactive health-seeking behaviors. For conditions carrying a high cosmetic burden (like adult-onset acne) or requiring prophylactic screening (like NMSC), these cohorts are significantly more likely to consult a dermatologist [44]. Consequently, mild or asymptomatic cases that might remain undiagnosed in lower socioeconomic strata are formally documented in highly educated cohorts, inflating the epidemiological odds ratio.

Conversely, the data demonstrated that higher educational attainment was associated with a decreased risk of psoriasis (OR = 0.90) [15]. Because moderate-to-severe plaque psoriasis is highly visible and physically debilitating, it is less susceptible to the ascertainment biases seen in mild acne; patients across all socioeconomic levels are eventually compelled to seek care. As an interpretative hypothesis, this negative correlation may reflect a genuine biological shielding effect mediated by lifestyle factors. Higher educational attainment frequently correlates with a lower prevalence of systemic comorbidities, such as obesity and chronic tobacco use, which are known drivers of the pro-inflammatory adipokines (e.g., TNF-α, IL-6) that sustain psoriatic inflammation [5]. Furthermore, higher education may correlate with the deployment of more adaptive stress-coping mechanisms, potentially dampening the neuroendocrine triggers of the disease [3]. However, due to the cross-sectional nature of the data, this proposed causal pathway remains an interpretative hypothesis requiring longitudinal validation.

### 4.3. Sex and Gender Differences

Precision dermatological epidemiology requires a demarcation between biological sex differences and sociocultural gender constructs. The Polish dataset highlighted a striking dimorphism, demonstrating that men faced substantially higher odds for both psoriasis (OR = 2.10) and tinea pedis (OR = 2.10) [15].

The markedly higher prevalence and clinical severity of psoriasis in men represent a complex interplay between gonadal sex hormones and systemic immune responses. Estrogens generally exert an immunomodulatory effect, often suppressing excessive Th1 and Th17 activation, whereas the androgenic hormonal environment in males interacts differently with the pathogenic IL-23/Th17 immune–inflammatory network [45].

However, while biological factors are critical, sociocultural behavioral norms may also serve as modifiers. As an interpretative hypothesis, the twofold increase in psoriasis odds among men could be partially attributed to delayed health-seeking behavior. Societal constructs surrounding masculinity may discourage proactive medical consultation, potentially leading men to delay seeking specialized care until skin lesions reach a more physically debilitating state.

Similarly, the elevated odds for tinea pedis (OR = 2.10) in men are likely intertwined with occupational exposome factors. Globally, men are statistically overrepresented in heavy industrial, agricultural, and construction roles that mandate the prolonged use of highly occlusive, non-breathable safety footwear [44]. This specific micro-environmental exposome is characterized by elevated temperatures and drastically increased relative humidity, providing a highly favorable biological incubator for the rapid colonization of dermatophytes. Thus, strict occupational norms translate directly into localized cutaneous pathology.

**Table 2:** Sociodemographic modifiers and interpretative hypotheses.

Variable	Finding (Polish Cohort)	Proposed Biological Mechanism	Interpretative Hypothesis (Sociocultural/Bias)
Urbanization	Increased AD (OR = 1.20) and NMSC (OR = 2.00)	AhR pathway activation via PM2.5/PAHs; UV–pollutant synergistic genotoxicity.	Higher NMSC rates partially reflect ascertainment bias due to superior urban access to dermoscopy screening.
Higher Education	Increased acne (OR = 1.50) and NMSC (OR = 1.40)	N/A (Unlikely to be a direct biological cause)	Ascertainment Bias: Greater health literacy and resources drive proactive cosmetic consultations and preventative oncological screenings.
Higher Education	Decreased psoriasis (OR = 0.90)	Mitigation of systemic inflammation via lower rates of comorbid obesity (reduced adipokines).	Protective Hypothesis: Correlation with adaptive stress coping mechanisms and adherence to healthier lifestyle profiles.
Male Sex/Gender	Increased psoriasis (OR = 2.10) and Tinea Pedis (OR = 2.10)	Distinct gonadal hormone interactions with the Th17 axis; thicker stratum corneum and higher baseline sebum.	Behavioral Hypothesis: Delayed diagnostic presentation leads to recording of more severe psoriasis cases; strict occupational footwear requirements drive tinea pedis.

## 5. Behavioral, Psychosocial, and Lifestyle Interventions in Dermatology

The formulation of the skin–brain–exposome axis represents a paradigm shift in contemporary dermatological science, moving the discipline toward an integrated model of neuroimmunological homeostasis. The large-scale 2023 Polish cohort demonstrated that effective psychosocial stress coping mechanisms were significantly associated with a reduced manifestation of distinct dermatological symptoms, including pruritus, burning, and redness (*p* < 0.001) [3]. Furthermore, the study established inverse associations between symptom prevalence and favorable lifestyle indicators: adequate sleep (OR = 0.84) and regular physical activity (OR = 0.86). A comprehensive summary of the skin–brain–exposome axis and these cohort findings across core dermatoses is provided in Table 3.

However, a fundamental methodological guardrail must be established: observational epidemiological associations detailing risk reduction do not equate to the therapeutic efficacy of targeted clinical interventions. To translate exposome theory into evidence-based therapeutics, we must contextualize these population-level survey data against the highest echelons of interventional evidence (meta-analyses and RCTs).

### 5.1. Psychotherapeutic Interventions: Efficacy and Evidence Grading

Psychotherapeutic interventions, including Mindfulness-Based Stress Reduction (MBSR) and Cognitive Behavioral Therapy (CBT), aim to cognitively intercept the stress–inflammation cycle.

Mindfulness and Meditation: In the context of atopic dermatitis (AD), a landmark randomized clinical trial (N = 107) evaluated an integrated online MBSR and self-compassion program. The intervention yielded statistically significant improvements compared to standard care, with the intervention group demonstrating a marked reduction in the Dermatology Life Quality Index (DLQI) at 13 weeks (between-group difference: −6.34; 95% CI, −8.27 to −4.41; *p* < 0.001) and a large standardized effect size (Cohen’s d = −1.06) [17]. However, in psoriasis, the evidence for MBSR remains conflicting regarding physical disease clearance. While early pilot studies and randomized trials showed promise [46,47,48,49], comprehensive meta-analyses indicate that mindfulness primarily improves psychological coping rather than generating sustained, objective reductions in the Psoriasis Area and Severity Index (PASI) [30,50].

Cognitive Behavioral Therapy (CBT) and Habit Reversal: CBT and Habit Reversal Therapy (HRT) directly target the destructive physical feedback loops characterizing chronic pruritus. A 2024 meta-analysis by Zhong et al. across six RCTs (N = 246) evaluating behavioral interventions in AD revealed a statistically significant reduction in overall eczema severity (correlation coefficient r = −0.39, *p* < 0.001) and scratching severity (r = −0.19, *p* = 0.017). Crucially, the interventions failed to exert any significant effect on the primary sensory intensity of the itch itself (r = −0.02, *p* = 0.840) [51]. This divergence illuminates the mechanism of action: CBT and HRT do not downregulate the neuroimmunological generation of pruritus, but rather physically intercept the pathological behavioral response (scratching), preserving the epidermal barrier and halting secondary inflammation. Similarly, an RCT (N = 168) evaluating internet-delivered CBT for AD showed it was non-inferior to clinician-guided therapy, yielding significant post-intervention improvements on the Patient-Oriented Eczema Measure (POEM) by 4.60 points (95% CI, 2.57–6.64) [26].

**Table 3:** Summary of the skin–brain–exposome axis across core dermatoses.

Dermatological Condition	Primary Pathomechanism (Skin–Brain–Exposome Axis)	Core Clinical Impact	Polish Cohort Findings (N = 27,000)
Atopic Dermatitis (AD)/Eczema	HPA axis dysfunction; neuropeptide (SP, CRH) release associated with mast cell degranulation; Th2/Th17 cytokine shift (IL-4, IL-13, IL-31); gut microbiome dysbiosis [5,10]	Acute exacerbation of pruritus intensity, localized desquamation, and transient erythema; severe disruption of sleep architecture [11].	Prevalence: 25.5%. Urbanization: Increased odds in cities >500 k inhabitants (OR = 1.20) [15].
Psoriasis	HPA axis hyperactivation; TNF-α, IL-17, and IL-22 upregulation driving systemic inflammatory cascade [19,20].	Rapid flare-ups of erythematous scaling and localized induration; accelerated keratinocyte hyperproliferation.	Prevalence: 5.0%. Gender: Higher odds in men (OR = 2.10). Education: Protective association from higher education (OR = 0.90) [15].
Acne Vulgaris	Local HPA-like axis activation; CRH-stimulated sebocyte lipogenesis; interactions with dietary exposome (e.g., high glycemic index) [5,31].	Increased sebum overproduction progressing to inflammatory papules, pustules, and comedones.	Prevalence: 32.7%. Education: Higher odds associated with advanced education, likely reflecting ascertainment bias (OR = 1.50) [15].
Rosacea	Autonomic neurovascular dysregulation; neurogenic inflammation via CGRP release causing microvascular dilation [5].	Acute vasomotor flushing, neurogenic stinging/burning, and transient centrofacial erythema; recent Mendelian randomization analyses suggest a potentially bidirectional relationship with psychiatric disorders [32].	Prevalence: 10.9%. Gender: Higher odds observed in women compared to men [15].
Hair Loss (General)	Stress-induced disruption of the anagen phase; potential premature apoptosis of hair follicle keratinocytes via SP/CRH; possible immune privilege collapse [6].	Reduction in hair density, patchy or diffuse shedding; profound negative impact on psychosocial identity.	Prevalence: 34.8%. Gender: Higher odds observed in women [15].
Chronic Pruritus (Transdiagnostic)	HPA/ANS hyperactivation; neurogenic sensitization; IL-31 and Substance P release [8,16].	Lowered threshold for itch perception; uncontrollable scratching bouts leading to secondary excoriations.	Stress Coping: Effective coping significantly associated with reduced general pruritus prevalence (*p* < 0.001) [3].

Note: SP = Substance P; CRH = corticotropin-releasing hormone; HPA = hypothalamic–pituitary–adrenal; ANS = autonomic nervous system; OR = odds ratio, CGRP = calcitonin gene-related peptide.

### 5.2. Lifestyle Modifications: Translating Observational Associations

Sleep Optimization: The Polish cohort’s observation that adequate sleep is associated with a reduced risk of skin symptoms (OR = 0.84) highlights a baseline homeostatic requirement [3]. Interventions addressing sleep, such as Cognitive Behavioral Therapy for Insomnia (CBT-I), have been shown to reduce systemic inflammatory biomarkers like interleukin-6 (IL-6) and tumor necrosis factor-alpha (TNF-α). While direct dermatological RCTs for these specific endpoints are currently limited, optimizing sleep architecture may offer a biologically plausible adjunctive pathway for managing inflammatory chronicity.

Physical Activity: The protective association between physical activity and dermatological health (OR = 0.86) aligns with the physiological understanding of exercise as a systemic anti-inflammatory mechanism [3]. In active dermatological disease, the interventional evidence is strongest for psoriasis, particularly when coupled with weight loss. A pivotal 20-week RCT evaluating diet and exercise in overweight patients with active plaque psoriasis found that 49.7% of patients in the active lifestyle intervention arm achieved a PASI-50 response (a 50% reduction in disease severity), compared to only 34.2% in the information-only control arm (*p* = 0.006) [52]. While this remains the most robust available RCT specifically evaluating structured physical activity and diet on PASI outcomes in psoriasis, its foundational findings have been subsequently corroborated by recent systematic reviews demonstrating that exercise interventions produce significant reductions in PASI scores across psoriatic patient populations [53]. For psoriasis, structured physical activity operates as a strong adjunctive intervention to decrease the systemic inflammatory burden of visceral adipokines.

Tobacco Cessation: Beyond psychological and physical factors, tobacco smoking represents a critical chemical and physiological stressor within the lifestyle exposome. Tobacco use is known to drive systemic inflammation by upregulating pro-inflammatory cytokines, such as TNF-α and IL-6, which are primary mediators in the pathogenesis of psoriasis and other stress-sensitive dermatoses [6,9,54]. Within the framework of the skin–brain–exposome axis, chronic tobacco use may act as an external environmental insult that exacerbates the neuroendocrine triggers of disease. Consequently, integrating smoking cessation into comprehensive dermatological care is essential for mitigating the cumulative inflammatory burden and enhancing the effectiveness of stress reduction strategies.

Microbiome and Dietary Modifications: The external and internal exposomes, particularly regarding diet and microbial flora, play a critical role in the management of acne vulgaris. High-glycemic-index diets are known to elevate insulin-like growth factor 1 (IGF-1), which exacerbates local sebocyte lipogenesis and comedogenesis. Consequently, transitioning to low-glycemic-index nutrition serves as a promising adjunctive strategy to decrease sebaceous gland overactivity. Furthermore, directly modulating the cutaneous microbiome has shown quantifiable benefits. A randomized controlled trial evaluating a topical probiotic formulation containing *Enterococcus faecalis* demonstrated significant improvements in investigator-assessed acne severity scores within two to six weeks of application. Together, these findings indicate that addressing both the nutritional exposome and localized dysbiosis may serve as useful adjuncts in comprehensive acne management.

A practical guide for clinicians regarding the clinical impact and evidence levels for the psychosocial and lifestyle interventions detailed above is presented in Table 4.

**Table 4:** Practical guide for clinicians: Psychosocial and lifestyle interventions.

Intervention Modality	Target Condition	Clinical Impact & Symptom Management	Narrative Level of Support
Cognitive Behavioral Therapy (CBT) & Habit Reversal	AD, Chronic Pruritus, Psoriasis	Disrupts the physical itch–scratch cycle; manages compulsive scratching and reduces psychological stigmatization; improves disease-specific QoL [26,40].	Best supported (Consistent evidence from RCTs/Meta-analyses for AD/Pruritus) Promising but limited (Psoriasis)
Mindfulness-Based Stress Reduction (MBSR)	Atopic Dermatitis, Psoriasis	Significantly improves health-related quality of life (DLQI) and subjective itch coping; modulates affective response to stress [17,30].	Best supported for QoL (AD) Mainly observational/Limited for physical clearance (Psoriasis)
Sleep Optimization	General Inflammatory Dermatoses	Hypothesis supports restoration of diurnal cortisol regulation and reduction in systemic pro-inflammatory cytokines (IL-6, TNF-a). Observational cohort supports protective association (OR = 0.84) [3].	Mainly observational/Mechanistic
Physical Activity	Psoriasis (especially with comorbid obesity), General	Acts as a systemic anti-inflammatory; reduces visceral adipokines. RCTs demonstrate improved PASI-50 response when combined with diet. Observational cohort supports protective association (OR = 0.86) [3,52].	Best supported (Psoriasis) Preliminary (AD)
Tobacco Smoking	Psoriasis, General Inflammatory Dermatoses	Mitigates systemic inflammation by reducing pro-inflammatory cytokines (TNF-α, IL-6); reduces the cumulative inflammatory burden on the cutaneous barrier.	Strong mechanistic and observational support (especially for Psoriasis).
Microbiome & Dietary Modification	Acne Vulgaris	Low-glycemic index diets decrease sebocyte lipogenesis [31]. Topical probiotics (e.g., *E. faecalis*) improve clinical severity [36].	Promising but limited

Note: DLQI = Dermatology Life Quality Index; PASI = Psoriasis Area and Severity Index; QoL = quality of life; OR = odds ratio; RCT = randomized controlled trial; AD = atopic dermatitis.

Limitations of the Review: While this manuscript provides a comprehensive conceptual integration, several methodological limitations must be acknowledged. First, as a narrative synthesis with a structured but non-exhaustive search strategy, this review inherently carries the potential for selection and confirmation bias compared to a strict PRISMA-compliant systematic review. Second, formal quality assessment tools (e.g., GRADE or SIGN criteria) were not uniformly applied to all cited mechanistic studies. While this review utilized a structured search strategy, we did not apply a formal numerical quality scoring tool to all cited mechanistic and observational studies, which may limit the comparability of certain findings. Finally, the population-level data heavily utilized herein relies on cross-sectional, self-reported survey methodologies. While these data establish robust statistical associations (e.g., between coping mechanisms and symptom reduction), they cannot definitively prove temporal causation regarding the initial neurobiological onset of the diseases. It must be underscored that while effective stress coping is robustly correlated with lower symptom prevalence, these cross-sectional findings represent a “snapshot” in time. Therefore, it remains unclear whether adaptive coping prevents the onset of dermatoses or if lower disease severity simply facilitates better psychological resilience.

To transition the skin–brain–exposome axis from theoretical modeling into precision psychodermatology, we propose the following framework and research directives:

1. Longitudinal Biomarker Tracking: Future research must prioritize prospective, longitudinal cohort studies that concurrently track validated psychometric scales with objective in vivo biological markers (e.g., continuous epidermal cortisol monitoring, serum IL-31/IL-17 levels, and transepidermal water loss) to firmly establish temporal causality between psychological stress spikes and subsequent inflammatory flares.
2. Implementation of Routine Psychosocial Screening (The Clinical Framework): The disconnect between objective skin clearance (PASI/SCORAD) and residual psychological trauma mandates a shift in routine dermatological care. We propose a “treat-to-target” clinical framework where dermatologists systematically administer validated tools like the Dermatology Life Quality Index (DLQI) [55] and the Patient Health Questionnaire-9 (PHQ-9) alongside physical examinations. To bridge the psychological–cutaneous gap, the primary recommendation is the formal adoption of an integrated care model, where “colocated behavioral health care” ensures that dermatologists and psychotherapists work in tandem to manage the neuroimmune chronicity of skin diseases.
3. Stratified Adjunctive Pathways: Utilizing this dual-screening framework, clinicians can stratify patients into targeted psychodermatological pathways. As a hypothesis-generating framework, patients presenting with low physical severity but disproportionately high DLQI/PHQ-9 scores might benefit from early triage toward CBT or behavioral interventions, serving as a conservative adjunctive step prior to considering escalation to heavier systemic therapies. In patients where psychological stress is identified as a primary “flare-trigger,” psychotherapeutic interventions like CBT or HRT should be introduced on par with traditional anti-inflammatory treatments, shifting from a supplementary role to a core component of the initial therapeutic strategy.

Ultimately, comprehensive dermatological care may benefit from broader integration of the underlying neuroendocrine and psychological exposome.

## 6. Conclusions

The epidemiology of dermatological disease represents a substantial global public health priority. As evidenced by the 89.5% lifetime prevalence of physician-diagnosed skin conditions in the Polish cohort, cutaneous morbidity is ubiquitous. This narrative synthesis has deconstructed the skin–brain–exposome axis, illustrating the plausible pathways through which psychological distress hyperactivates the HPA axis and peripheral neurogenic inflammation to drive conditions like atopic dermatitis, psoriasis, and acne [5,11]. Importantly, the integration of macroscopic sociodemographic modifiers—such as urban pollutant exposure and the diagnostic paradoxes tied to educational attainment—demonstrates that biological vulnerabilities are continuously shaped by the external exposome. To address these multidimensional risks, effective stress reduction transcends generic lifestyle advice; it requires structured techniques such as CBT/HRT to disrupt the physical itch–scratch cycle, Mindfulness-Based Stress Reduction (MBSR) to improve disease-specific quality of life, and targeted relaxation modalities, including music therapy, for the immediate symptomatic relief of acute pruritus. The clinical trial data suggests that psychotherapeutic interventions (CBT, MBSR) do not serve as standalone immunomodulators, but rather may serve as useful adjuncts, particularly for QoL and behavioral symptom loops that disrupt the itch–scratch cycle and restore psychological quality of life [17,26].

## Data Availability

No new data were created or analyzed in this study.

## Abbreviations

The following abbreviations are used in this manuscript:

AA	Alopecia areata
AD	Atopic Dermatitis
AhR	Aryl hydrocarbon receptor
ANS	Autonomic Nervous System
CBT	Cognitive Behavioral Therapy
CBT-I	Cognitive Behavioral Therapy for Insomnia
CGRP	Calcitonin gene-related peptide
CI	Confidence Interval
CNS	Central Nervous System
DALYs	Disability-adjusted life years
DLQI	Dermatology Life Quality Index
GBD	Global Burden of Disease
HPA	Hypothalamic–pituitary–adrenal
HRT	Habit Reversal Therapy
IGF-1	Insulin-like growth factor 1
IL	Interleukin
MBSR	Mindfulness-Based Stress Reduction
MeSH	Medical Subject Headings
NGF	Nerve growth factor
NK-1	Neurokinin-1
NMSC	Non-melanoma skin cancer
NO2	Nitrogen dioxide
OR	Odds Ratio
PAHs	Polycyclic aromatic hydrocarbons
PASI	Psoriasis Area and Severity Index
PHQ-9	Patient Health Questionnaire-9
PM2.5/PM10	Particulate matter
POEM	Patient-Oriented Eczema Measure
QoL	Quality of Life
RCTs	Randomized controlled trials
SCFA	Short-chain fatty acid
SCORAD	SCORing Atopic Dermatitis
SMD	Standardized Mean Difference
SP	Substance P
TEWL	Transepidermal water loss
Th1/Th2/Th17	T-helper cells
TNF-α	Tumor necrosis factor-alpha
UV	Ultraviolet
VOCs	Volatile organic compounds
